# Clustering by antigen-presenting genes reveals immune landscapes and predicts response to checkpoint immunotherapy

**DOI:** 10.1038/s41598-023-28167-1

**Published:** 2023-01-18

**Authors:** Xutong Gong, Rachel Karchin

**Affiliations:** 1 Department of Biomedical Engineering, Johns Hopkins University, 217A Hackerman Hall, 3400 N. Charles St., Baltimore, MD 21218 USA; 2 Institute for Computational Medicine, Johns Hopkins University, Baltimore, MD 21218 USA; 3 Department of Oncology, Johns Hopkins Medicine, Baltimore, MD 21287 USA

**Keywords:** Oncology, Computational biology and bioinformatics, Computational models, Immunology, Adaptive immunity, Antigen processing and presentation, Immune evasion, Immunotherapy, Cancer, Tumour immunology

## Abstract

Immune checkpoint blockade (ICB) has demonstrated efficacy by reinvigorating immune cytotoxicity against tumors. However, the mechanisms underlying how ICB induces responses in a subset of patients remain unclear. Using bulk and single-cell transcriptomic cohorts of melanoma patients receiving ICB, we proposed a clustering model based on the expression of an antigen-presenting machinery (APM) signature consisting of 23 genes in a forward-selection manner. We characterized four APM clusters associated with distinct immune characteristics, cancer hallmarks, and patient prognosis in melanoma. The model predicts differential regulation of APM genes during ICB, which shaped ICB responsiveness. Surprisingly, while immunogenically hot tumors with high baseline APM expression prior to treatment are correlated with a better response to ICB than cold tumors with low APM expression, a subset of hot tumors with the highest pre-ICB APM expression fail to upregulate APM expression during treatment. In addition, they undergo immunoediting and display infiltration of exhausted T cells. In comparison, tumors associated with the best patient prognosis demonstrate significant APM upregulation and immune infiltration following ICB. They also show infiltration of tissue-resident memory T cells, shaping prolonged antitumor immunity. Using only pre-treatment transcriptomic data, our model predicts the dynamic APM-mediated tumor-immune interactions in response to ICB and provides insights into the immune escape mechanisms in hot tumors that compromise the ICB efficacy. We highlight the prognostic value of APM expression in predicting immune response in chronic diseases.

## Introduction

Immune checkpoint blockade (ICB) has shown efficacy in tumor rejection and has improved patient survival in many tumor types by blocking the receptor-ligand interactions of inhibitory immune checkpoints^1^. The response to therapy is closely related to the antigen presenting machinery (APM), in which antigens are processed and loaded onto the major histocompatibility complex (MHC) to facilitate T-cell recognition and immune clearance^2^. Interactions between the peptide-MHC complex and T cell receptor (TCR) are key for successful T cell priming and differentiation into effector cells with anti-tumor cytotoxicity^3^. Recent studies have focused on the identification of neoantigens present on tumors that trigger an immune response^4^. Strong APM gene expression has been shown to facilitate immune infiltration and enhance tumor immunogenicity^5^.

Despite the fact that ICB reinvigorates immune cells and enhances antitumor cytotoxicity, many patients remain unresponsive to ICB^6^. Both intrinsic and extrinsic mechanisms in the tumor microenvironment prevent immune recognition and the formation of prolonged antitumor immunity. For instance, malignant cells with low mutational burden are less immunogenic and cannot be efficiently targeted by immune cells as non-self^7^. Immune selection resulting from TCR neoantigen binding can lead to tumor evolution and subsequent loss of expression of the most immunogenic neoantigens^8^. In recent years, significant efforts have been made to convert cold tumors into hot tumors by increasing tumor immunogenicity and enhancing the neoantigen specificity of T cells^9,10^.

In contrast, while high immunogenicity generally correlates with better prognosis, a subset of hot tumors remain unresponsive to therapy^11^. The mechanisms that drive the immune escape of immunogenic tumors have not been fully elucidated. Clinical metrics, including mutational load and immune checkpoint expression, only demonstrate prognostic value in some patients^7,10^. Understanding how ICB triggers an effective immune response in the tumor microenvironment (TME) will guide clinical decision-making and improve the survival benefits of ICB. We propose that examining APM expression patterns and the underlying immune landscape will provide insights into how APM shapes the responsiveness to therapy. Using three bulk RNA-seq cohorts^8,12,13^ and one single-cell RNA-seq (scRNA-seq) cohort^14^, we studied how APM shape tumor immunogenicity and response to ICB. Using hierarchical clustering, we identified four APM clusters with distinct immune landscapes. CD4 + and CD8 + T cell subsets were differentially enriched across clusters, potentially shaping patient response to therapy. We also investigated the dynamic regulation of APM expression and immune cell infiltration after the onset of ICB. Furthermore, the APM clustering model demonstrated prognostic value for melanoma and outperformed conventional clinical metrics in predicting patient survival. Taken together, our APM clustering model uncovered mechanisms contributing to immune escape and the lack of response to ICB.

## Results

### APM clustering predicts response to ICB

To dissect the role of APM expression in shaping immunogenicity, we identified genes in the antigen presentation pathway that were previously confirmed to be upregulated upon infection or chronic inflammation, including human leukocyte antigen (HLA) class I and class II genes, proteasome genes, and genes facilitating antigen processing and loading^15,16^. However, while genes participating in MHC I antigen presentation are ubiquitously expressed, their expression are in general significantly higher in immune cells than tumor cells^17^. Hence, to investigate how MHC I-mediated tumor antigen presentation shapes the immune landscape, correction of expression values from bulk RNA-seq data is needed to reflect tumor-specific expression levels more accurately. On the other hand, since MHC II-mediated antigen presentation selectively takes place in immune cells, we did not apply expression correction to MHC II and associated genes. Using an scRNA-seq cohort^14^, we computed a normalization factor with respect to the expression level of each HLA I and associated genes (Methods). The expression of individual genes was corrected using a normalization factor and tumor purity. To evaluate the performance, we applied the correction to patient-level pseudobulk expression profiles of all cells, which mimicked bulk RNA-seq expression profiles. After gene expression correction, the overall pseudobulk gene expression levels were no longer differently distributed compared to those of malignant cells (Supplementary Fig. S1A-B). Consequently, we employed the same correction procedure for bulk RNA-seq expression profiles for all subsequent analyses.

**Figure 1:**
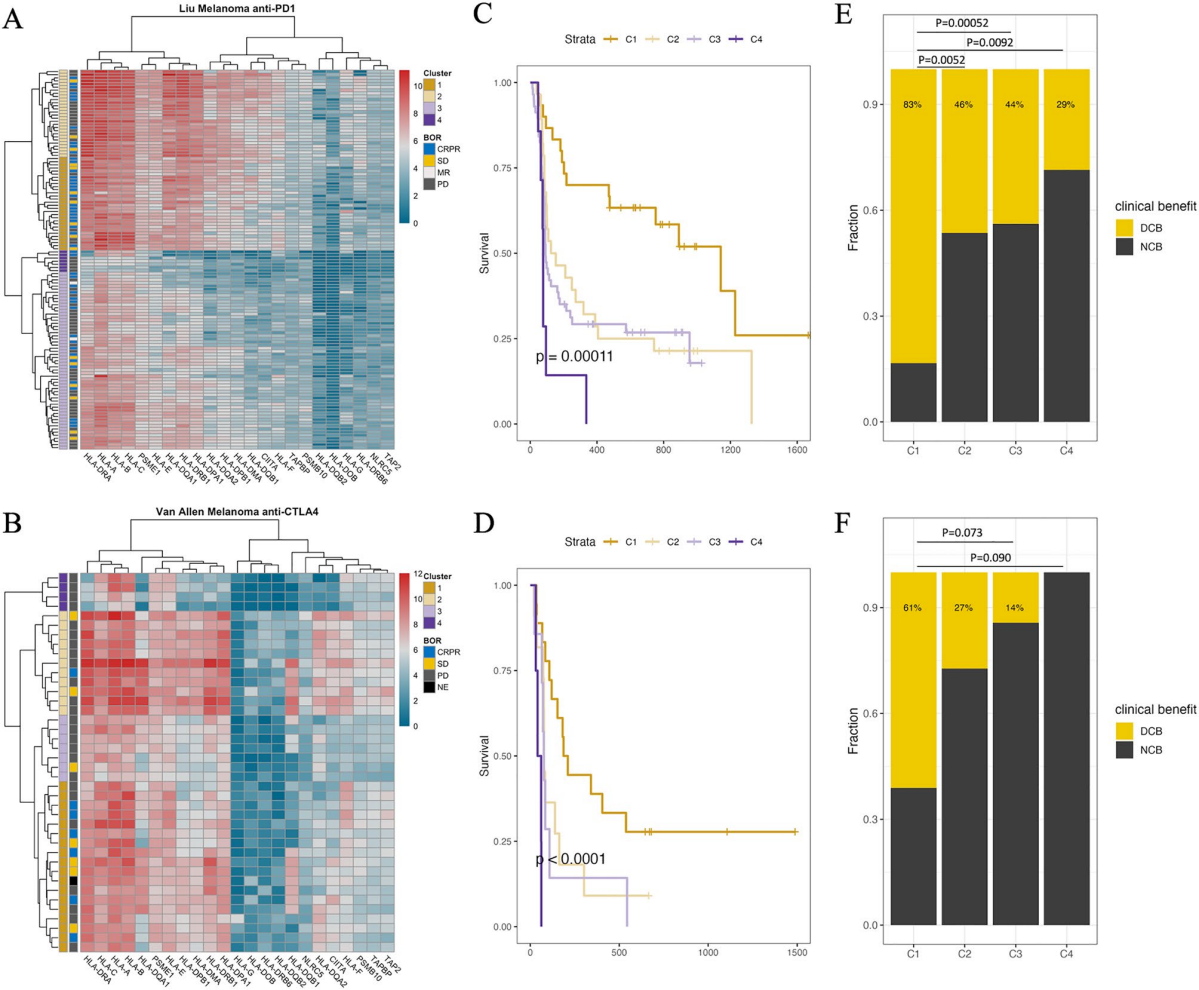
Unsupervised hierarchical clustering on APM genes correlates with patient survival. (**A,B)** Independent clustering in the Liu melanoma anti-PD1 cohort (n = 122) (**A**) and Van Allen anti-CTLA4 cohort (n = 40) (**B**) (discovery cohorts) using the trained APM gene signature. pheatmap function in pheatmap version 1.0.12 (https://www.rdocumentation.org/packages/pheatmap/versions/1.0.12) was used to generate the heatmaps. Each row represents a patient, and each column represents a gene. Gene expression is represented as log transformed transcripts per million (TPM) reads. *BOR* best overall response, *CRPR* complete/partial response, *SD* stable disease, *MR* mixed response, *PD* progressive disease. (**C,D**) Progression-free survival (PFS) stratified by the APM clusters in the Liu cohort (C1: n = 30, C2: n = 28, C3: n = 57, C4: n = 7) (**C**) and Van Allen cohort (C1: n = 18, C2: n = 11, C3: n = 7, C4: n = 4) (**D**) represented as Kaplan–Meier curves. P-value was computed by Log-rank test. (**E–F**) Proportion of patients with DCB versus NCB across the APM clusters in the Liu cohort (**E**) and Van Allen cohort (**F**). Percentage indicates fraction of patients with DCB within the cluster. Significance was calculated by two-sided Fisher’s exact test. *DCB* durable clinical benefit, *NCB* no clinical benefit.

We first investigated the association between the pre-treatment APM gene expression patterns and patient survival in two melanoma cohorts receiving anti-PD1 (n = 122) and anti-CTLA4 (n = 40) treatment^12,13^. Overall, the expression of individual APM genes was correlated with patient survival (Supplementary Fig. S2A-B). We used both cohorts as discovery cohorts to identify the most prognostic combination of genes in a forward-selection-based process (Methods). A four-cluster model generated by hierarchical clustering on the proposed APM signature (Fig. 1A,B) significantly predicted progression-free survival in both cohorts (Log-rank test, Liu cohort: p = 6.0e−4; Van Allen cohort: p = 1.2e−4) (Fig. 1C,D). C1 and C2 showed higher HLA class I, class II, and proteasome gene expression than C3 and C4 did. C1 showed the strongest correlation with durable clinical benefit (DCB), whereas C4 showed the strongest correlation with no clinical benefit (NCB) (%DCB within cluster, Liu C1: 83%, C2: 46%, C3: 44%, C4: 29%; Van Allen C1: 61%, C2: 27%, C3: 14%, C4: 0%) (Fig. 1E,F). The results demonstrated that the APM clusters significantly stratified patient survival and response to anti-PD1 and anti-CTLA4 treatments, the two most frequently used immune checkpoint blockade treatments.

### APM expression shapes distinct immune landscapes

**Figure 2:**
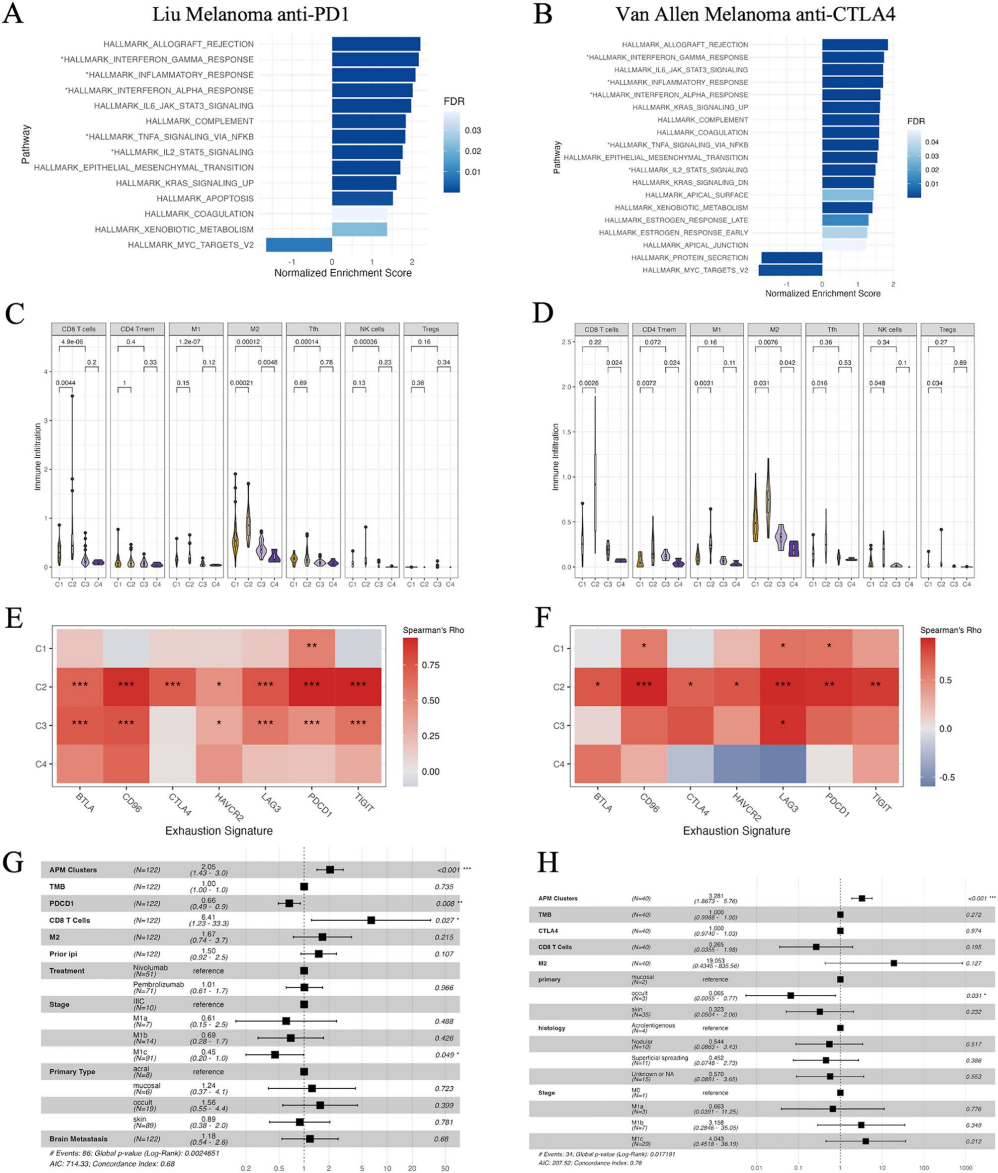
APM signature reveals immune landscape. (**A,B**) Pathway enrichment analysis computed by GSEA in the Liu cohort (**A**) and Van Allen cohort (**B**). The hallmark gene category annotated by MSigDB was used. Pathways with positive normalized enrichment scores (NES) were enriched in C1 and C2 tumors, and those with negative NES were enriched in C3 and C4 tumors. Asterisks denote proinflammatory pathways. Colors represent false discovery rate (FDR). Pathways with FDR $\le$ 0.05 are shown. (**C,D**) Immune infiltration inferred by immune cell deconvolution in the Liu cohort (**C**) and Van Allen cohort (**D**). Boxes in the violin plots represent interquartile ranges and vertical lines represent 5th–95th percentile ranges. Significance between the clusters was computed by Wilcoxon rank-sum test. *Tmem* memory T cells, *M1* macrophage 1, *M2* macrophage, *Tfh* follicular helper T cells, *NK* natural killer cells, *Tregs* regulatory T cells. (**E,F**) Spearman correlations between CD8 T cell fractions and expression of the exhaustion signature across C1–C4 in the Liu cohort (**E**) and Van Allen cohort (**F**). Spearman’s rank correlation was used to calculate correlation coefficients and p-values. Asterisks denote significant p-values adjusted by Benjamini & Hochberg (BH) method (*p < 0.05; **p < 0.01; ***p < 0.001). (**G,H**) Multivariate Cox proportional hazards model of APM clusters and clinical characteristics in the Liu cohort (**G**) and Van Allen cohort (**H**). APM clusters (C1 = 1, C2 = 2, C3 = 3, C4 = 4), tumor mutational burden (TMB), *PDCD1* expression, *CTLA4* expression, CD8 T cell fractions, M2 fractions, prior ipilimumab (ipi) treatment (true = 1, false = 0), and brain metastasis (true = 1, false = 0) are continuous variables. Hazard ratios (HR), 95% confidence intervals (CI), and p-values are shown.

Next, we examined the immune characteristics associated with each APM cluster. Gene set enrichment analysis (GSEA) showed that proinflammatory pathways were significantly enriched in C1 and C2 compared to C3 and C4 in both the Liu anti-PD1 and Van Allen anti-CTLA4 cohort (Fig. 2A,B). This suggests that C1 and C2 are immunogenically active, whereas C3 and C4 are immunologically inactive. Moreover, C2 showed the highest APM gene expression and immune infiltration, while C4 showed the lowest (Figs. 1A,B, 2C,D). Of note, tumor mutational burden (TMB) was not associated with APM expression or immune infiltration across the clusters (Supplementary Fig. S3A-B).

During ICB treatment, the reinvigoration of antitumor immune cells enhances tumor rejection^1^. Interestingly, among the immunogenically active C1 and C2, C2 was associated with worse survival, suggesting that there might be a means of immune escape other than a lack of immunogenicity. We next investigated tumor-intrinsic and extrinsic mechanisms that might explain the prognostic discrepancy between C1 and C2. While C2 had the highest APM expression (Supplementary Fig. S3C-D), GSEA between C1 and C2 revealed that C2 had upregulated pro-tumorigenic pathways, including epithelial-mesenchymal transition, KRAS signaling, and IL6-JAK-STAT3 signaling (Supplementary Fig. S3E-F). C2 tumors also showed significantly higher enrichment of angiogenesis, hypoxia, proliferation, reactive oxygen species (ROS) pathway, and TGF-$\upbeta$ signaling, processes known to suppress the immune response and facilitate tumor progression^18–20^ (Supplementary Fig. S3G). We further hypothesized that the infiltrated immune cells in C2 tumors might be associated with immune dysfunction. Supporting our hypothesis, CD8 + T cell infiltration in C2 tumors demonstrated a significant correlation with an immune exhaustion signature^21,22^ (Fig. 2E,F). In contrast, C1 tumors showed weak to no correlation between CD8 T cells and the exhaustion signature, despite high immune infiltration. Furthermore, we found that low *PDCD1* expression was associated with better prognosis in C1-C2 tumors, whereas it was not correlated with patient survival in C3-C4 tumors (Supplementary Fig. S3H-I). PD-1 expression evaluated using immunohistochemistry (IHC) is a frequently used prognostic factor^23^. While immunogenically hot tumors generally displayed higher *PDCD1* expression (Supplementary Fig. S3J), we suggest that the immune cells with lower *PDCD1* expression in hot tumors may be more responsive to ICB. Together, these results show that C3 and C4 tumors might undergo immune escape through a lack of immune infiltration because of low APM expression. In comparison, while both C1 and C2 had high levels of immune infiltration and better patient prognosis, C2 tumors with the highest APM expression likely underwent tumor escape due to high selective pressure by upregulating pro-tumor mechanisms and co-inhibitory molecules to suppress the immune activity and facilitate tumor progression, resulting in a dysfunctional immune landscape and ineffective tumor clearance during ICB treatment.

Next, we aimed to simultaneously dissect the effects of APM clusters and potentially interacting covariates on patient survival, including immune characteristics, clinically defined tumor subtypes and stages, and other clinical predictors, using a multivariate Cox proportional hazards model (Fig. 2G,H). In both cohorts, APM clusters demonstrated the most significant association with patient prognosis after adjusting for various tumor stages and histology (Cox proportional-hazards, Liu cohort: HR = 2.05, p < 0.001, Van Allen cohort: HR = 3.28, p < 0.001). Other commonly used clinical metrics, including TMB, *PDCD1* expression, and CTLA4 expression, were not strongly associated with patient survival. In addition, while the infiltration levels of CD8 + T cells and M2 correlated strongly with APM expression, they did not show a significant correlation with patient survival. Prior anti-CTLA4 (ipilimumab) treatment in the Liu anti-PD1 cohort also did not abolish the prognostic value of APM clusters on patient survival.

### APM upregulation is critical for optimal response to ICB

**Figure 3:**
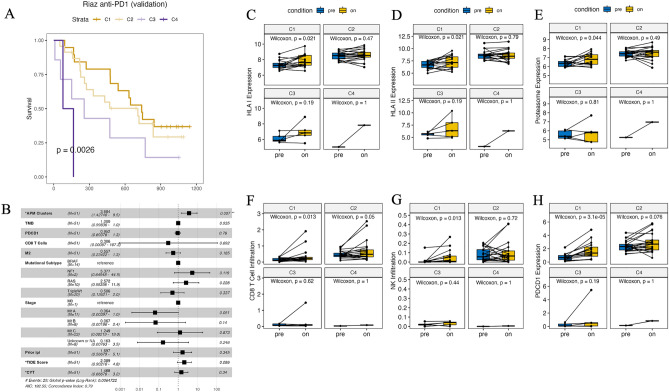
Differential regulation of APM and immune signatures across APM clusters during ICB. (**A**) Overall survival (OS) stratified by the predicted clusters (C1: n = 19, C2: n = 23, C3: n = 7, C4: n = 2) in the Riaz melanoma anti-PD1 cohort (n = 51, validation cohort) represented as Kaplan–Meier curves. P value was computed by Log-rank test. (**B**) Multivariate Cox proportional hazards model of APM clusters and clinical characteristics. APM clusters (C1 = 1, C2 = 2, C3 = 3, C4 = 4), tumor mutational burden (TMB), *PDCD1* expression, CD8 T cell fractions, M2 fractions, prior ipi treatment (true = 1, false = 0), CYT, and the TIDE scores are continuous variables. Asterisks denote published signatures for predicting immunogenicity and prognosis to ICB. Hazard ratios (HR), 95% confidence intervals (CI), and p-values are shown. (**C–E**) HLA class I (**C**), class II (**D**), and proteasome expression (**E**) in pre-treatment tumors and matched on-treatment tumors across the clusters (C1: n = 16, C2: n = 21, C3: n = 5, C4: n = 1, excluding cases without on-treatment samples). Boxes in the boxplots represent interquartile ranges and vertical lines represent 5th–95th percentile ranges. Significance was computed by Wilcoxon signed-rank test. (**F,G**), As in (**C–E**) but for CD8 T cells (**F**) and NK cells (**G**). (**H**) As in (**C–E**) but for *PDCD1* expression.

We then assessed whether the APM clustering model had prognostic value when applied to another melanoma cohort that received ICB. A multinomial logistic regression classifier was trained on the APM expression data and clustering results from both discovery cohorts, which achieved high training accuracy (mean micro-AUC = 0.98) (Methods). The classifier was then applied to an independent melanoma anti-PD1 validation cohort from Riaz et al.^8^ (n = 51), and the predicted clusters significantly stratified patient overall survival (log-rank test, p = 0.0026) (Fig. 3A). Consistent with the previous APM and immune characterization of the clusters, C1 and C2 had higher APM expression with an enrichment of proinflammatory pathways than C3 and C4 (Supplementary Fig. S4A-B). To further evaluate the prognostic value of our APM model, we compared its performance to two widely used frameworks to evaluate immunogenicity and prognosis to ICB: cytolytic score (CYT)^24^ as the log average expression of GZMA and PRF1, and Tumor Immune Dysfunction and Exclusion (TIDE) score^25^. While CYT did not correlate with patient survival, both the APM clusters (HR = 1.6, p = 0.036) and TIDE score (HR = 1.5, p = 0.039) showed comparable performance (Supplementary Fig. S4C). Interestingly, when the clinical characteristics, including TMB, *PDCD1* expression, CD8 T cell or M2 infiltration, mutational subtype, tumor stage, and prior ipilimumab treatment, were included as covariates in a multivariate Cox proportional hazards model, TIDE score no longer was no longer predictive of patient survival (Fig. 3B). In contrast, the prognostic value of APM clustering was not abolished by these clinical characteristics (Cox proportional hazards, HR = 3.68, p = 0.007) (Fig. 3B). As discussed by Jiang et al., TIDE had more limited application in tumors that progressed from prior ICB. Consequently, as 26 patients developed resistance to ipilimumab before receiving anti-PD1 therapy in the Riaz cohort, TIDE showed more compromised performance after adjusting for the interacting covariates. On the other hand, the APM clustering model was resistant to the covariates. Together, while TMB and immune checkpoint expression are among the most used clinical metrics for immunotherapy administration and prediction of response^26^, our results highlight the potential wide application of the APM model for predicting prognosis in ICB-naïve or prior ICB-progressed melanoma of various subtypes and stages.

Next, we evaluated the differential expression of APM genes between pre- and on-treatment tumor pairs. Pre-treatment HLA and immunoproteasome expression were significantly upregulated in the DCB group but not in the NCB group (Supplementary Fig. S4D-F). Interestingly, the predicted C1 tumors also showed significant APM upregulation (Wilcoxon signed-rank test, p = 0.016, p = 0.016, and p = 0.044 for HLA class I, class II, and immunoproteasome genes, respectively) during ICB (Fig. 3C–E). While the tumors from C2 demonstrated the highest APM expression in pre-treatment tumor samples, they displayed no upregulation in APM expression during treatment.

Furthermore, C1 tumors showed uniquely increased infiltration of CD8 + T cells (p = 0.013) and NK cells (p = 0.013) (Fig. 3F,G). In addition, *PDCD1* expression was upregulated in C1 (p = 3.1e-5), suggesting the reactivation of immune activity in response to anti-PD1 ICB (Fig. 3H). We also confirmed that the DCB group showed increased immune cell infiltration and elevated *PDCD1* expression, which was not observed in the NCB group (Supplementary Fig. S4G-I). Our results highlight the importance of APM upregulation during ICB to facilitate T cell infiltration for tumor rejection and prolonged survival.

### Differential enrichment of malignant and immune profiles across APM clusters

**Figure 4:**
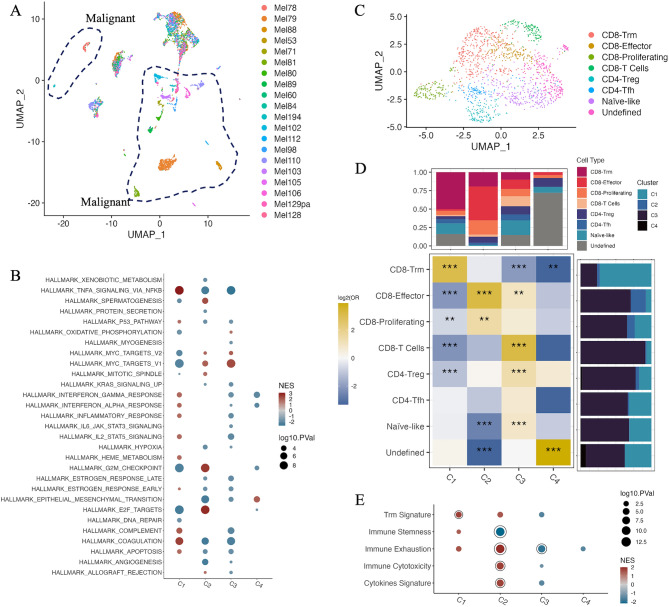
Biological processes of various cell types across APM clusters. (**A**) UMAP analysis of all cells (n = 5235) across 20 patients in the Jerby-Arnon scRNA-seq cohort. (**B**) Pathway enrichment analysis in malignant cells across APM C1-C4 computed by GSEA. The hallmark gene category annotated by MSigDB was used. Asterisks denote proinflammatory pathways. Pathways with FDR < 0.01 are shown. Colors represent normalized enrichment scores (NES), and dot sizes represent log10 (BH-adjusted p-value). (**C**) UMAP analysis of the T cell population. The cluster annotations were defined based on canonical and subset-selective markers between clusters. (**D**) Differential enrichment of T cell subsets across APM C1-C4. Odds ratios (OR) and p-values were calculated by two-sided Fisher’s exact test. ggplot function in ggplot2 version 3.4.0 (https://ggplot2.tidyverse.org) was used to generate the heatmap. Color scale in the heatmap represents log2 (OR). Asterisks denote significant BH-adjusted p-values adjusted (*p < 0.05; **p < 0.01; ***p < 0.001). Bar plots show fractions of cell population within a cluster or cell type as indicated. (**E**) Functional enrichment analysis in the T cell population across APM C1-C4. Pathways with FDR < 0.25 are shown. Circles denote FDR < 0.05. Immune signatures are provided in Supplementary Table S6.

To dissect the roles of various cell types in shaping APM clusters, we performed dimensionality reduction on all cells from the Jerby-Arnon scRNA-seq cohort^14^ (n = 20) through uniform manifold approximation and projection (UMAP) analysis (Supplementary Fig. S5A). Consistent with previous studies^27^, there was more heterogeneity in malignant cells across patients than in non-malignant cells, as malignant cells from different tumors of origin formed non-overlapping clusters (Fig. 4A, Supplementary Fig. S5B). Next, we predicted the APM clusters of 20 patients from the pseudobulk patient-level expression profiles using the same multinomial logistic regression classifier (Methods). Consistent with our characterization of APM clusters, C1 and C2 showed higher APM expression than C3 and C4 (Supplementary Fig. S5C). Interestingly, all post-immunotherapy-resistant patients were classified as having APM C3 (Supplementary Table S1). Malignant cells across APM C1-C4 demonstrated distinct enrichment of cancer hallmark pathways (Fig. 4B). Among them, C1 tumors are associated with TNF-$\alpha$ signaling via NF-$\kappa$B, C2 with G2M checkpoint and E2F targets, C3 with MYC targets, and C4 with epithelial-mesenchymal transition (EMT).

Next, we investigated the immune landscape across APM clusters with a specific focus on T cells, as they can directly target malignant cells. CD4 + and CD8 + T cell populations were clustered again to obtain more refined T cell subsets (Fig. 4C, Supplementary Fig. S5D). Interestingly, CD8 + tissue-resident memory (Trm) cells were significantly enriched in C1, and CD8 + effector T cells and proliferating T cells were enriched in C2, potentially contributing to enhanced antitumor immunity upon ICB (Fig. 4D). In contrast, C3 and C4 showed a lack of proinflammatory T cells but instead had an enrichment in CD4 + Tregs and naïve-like cells. Furthermore, GSEA of CD8 + T cells across the APM clusters showed that C1 upregulated the Trm signature, while C2 upregulated immune cytotoxicity, exhaustion, and cytokine signatures^28–31^ (Fig. 4E). Taken together, these results confirmed that C3 and C4 had little infiltration of T cells with direct cytotoxicity against tumors. In contrast, while the highest APM-expressing C2 had a cytotoxic but dysfunctional immune landscape, C1 with a particular enrichment of Trm cells could shape prolonged anti-tumor immunity upon ICB.

## Discussion

Functional antigen-presenting machinery is critical for eliciting immune-based tumor rejection. We and others have previously reported a strong association between MHC molecule levels and tumor immunogenicity^5,32^. In this study, we proposed an APM gene signature and identified four APM clusters using hierarchical clustering that were associated with distinct patient prognoses. We also characterized the underlying immune landscape associated with the APM clusters using bulk RNA-seq and scRNA-seq expression data. Using pre-treatment melanoma transcriptomic data, the APM clustering model predicted prognosis in patients who were either treatment-naïve or progressed from prior treatments. Our results highlight the broad application among patients with a variety of treatment backgrounds.

ICB disrupts receptor-ligand interactions of immune checkpoints to unleash immune cells^33^. Hence, higher levels of tumor-infiltrating lymphocytes (TILs), in general, could lead to better antitumor immunity upon ICB. However, some tumors with high baseline immunogenicity remain unresponsive to ICB^11,34^. Surprisingly, while the functional antigen presenting machinery is critical for anti-tumor immune cytotoxicity, we identified a subset of hot tumors (C2) with the highest APM expression associated with immune exhaustion and suboptimal patient prognosis. Multiple studies have identified populations of terminally differentiated TILs that cannot re-elicit full cytotoxicity even in the presence of immune checkpoint inhibitors. We propose that the chronic antigen stimulation owing to enhanced antigen presentation in C2 might lead to the upregulation of exhaustion programs to dampen immune cytotoxicity, which is a natural response to prevent tissue damage and autoimmunity^35^. Consequently, the infiltrating immune cells in C2 tumors likely exhibit minimal plasticity and diminished functional capacity. Our results emphasize that the functional states of immune cells, rather than absolute immune infiltration, are better prognostic predictors of the response to ICB in hot tumors.

Peptide-MHC and TCR interactions are critical for triggering the activation and differentiation of CD8 cytotoxic T cells and CD4 T helper cells^3^. Hence, the upregulation of APM genes likely correlates with increased neoantigen presentation, leading to immune infiltration and more favorable clinical outcomes. Using pre-treatment APM expression data, the APM model predicted tumors (C1) that could simultaneously upregulate APM expression and anti-tumor immune infiltration following treatment, which shaped the sensitivity to ICB. Interestingly, while C2 tumors had the highest pre-treatment APM expression, they did not show APM upregulation on-treatment. It is possible that they developed acquired resistance to therapy through tumor immunoediting because of strong immune-selective pressure. In contrast, C3 and C4 tumors likely develop intrinsic resistance due to low antigen presentation^11^. Consequently, while they could also upregulate APM genes, this upregulation might not efficiently compensate for their low pre-treatment APM expression.

We also characterized the enrichment of various T-cell subsets across APM clusters. Interestingly, CD8 + Trm cells were uniquely up-regulated in C1. Recent studies have reported that neoantigen-specific TILs mostly consist of Trm cells^36^. High Trm infiltration is also correlated with better prognosis^29^. We propose that CD8 + Trm cells in C1 tumors directly target malignant cells through interactions between neoantigen-specific TCRs and neoantigens present on MHC molecules. In contrast, the enrichment of CD8 + effector T cells and proliferating T cells in C2 tumors mediated by high APM expression might cause a strong inflammatory response through the secretion of cytokines such as IFN-$\upgamma$^37^. Although these cytokines can suppress tumor growth and induce apoptosis, they also lead to T cell exhaustion, compromising the efficacy of ICB^38^.

Our study had several limitations. First, we averaged all patients in the scRNA-seq cohort to calculate the normalization factors of APM genes. However, different types of non-malignant cells, such as infiltrating T and stromal cells, may express different levels of APM genes. Although multiple cell-type deconvolution algorithms have been proposed^39^, it remains challenging to infer all non-malignant cell types from bulk RNA-seq expression data. Second, we used the signature of seven housekeeping genes to correct for batch effects across cohorts. While these housekeeping genes generally have stable expression, there might exist variations in expression across disease conditions or tissue types, potentially restricting the generalizability of the APM clustering model to other cancer types. Despite these limitations, we demonstrated the prognostic value and practicability of APM clustering in melanoma patients. The model takes the pre-treatment transcriptome data of only 23 APM genes as input and can predict APM differential expression and immune infiltration following ICB, which strongly correlates with survival benefits. Additionally, our model identified a subset of hot tumors with the highest pre-treatment APM expression that were resistant to therapy through tumor immunoediting and immune exhaustion. We expect that our findings will be validated using larger cohorts with more tumor types to test the generalizability of our conclusions. While we investigated how APM shapes the responsiveness to therapy in cancer, our framework should be meaningful for studies of chronic disease and may be critical for understanding therapeutic resistance mechanisms. More generally, our feature selection pipeline might be useful for identifying other gene signatures from genome-wide or candidate genes as biomarkers for biological functions and patient prognosis.

## Methods

### Study cohort and data acquisition

We reviewed the literature and identified cohorts with available whole-exome sequencing (WES), bulk RNA-seq, and clinical information for more than 40 patients. The Liu melanoma anti-PD1^12^ and Van Allen melanoma anti-CTLA4^13^ cohorts were used to select the APM gene signature. The Riaz melanoma anti-PD1 cohort^8^ was used to evaluate the predictive power of the APM four-cluster model. The Jerby-Arnon scRNA-seq cohort^14^ was used to investigate differential enrichment of various cell types and their regulation of biological processes.

Clinical information for the Liu anti-PD1 (n = 122, including 48 patients progressed from prior ipilimumab treatment) and Van Allen anti-CTLA4 (n = 42) cohorts was obtained from Liu et al.^12^ and Van Allen et al.^13^, respectively. Pre-treatment raw sequencing data were obtained from dbGaP under accession phs000452.v3.p1 and phs000452.v2.p1 for the Liu and Van Allen cohorts, respectively. Two patients (SRR3083584 and SRR3083781) were excluded from the Van Allen cohort because of insufficient coverage at the Human Leukocyte Antigen (HLA) loci. The mapped raw counts were computed using FeatureCounts^40^. Transcripts per million (TPM) were calculated to represent gene expression.

For validation, clinical information of the Riaz anti-PD1 cohort (n = 51, including 26 patients progressed from prior ipilimumab treatment) was obtained from Riaz et al.^8^. On-therapy samples were collected from the same site between days 23–29 after the first dose of therapy. Transcriptomic data in mapped raw counts for pre- and on-treated tumor samples were obtained from the Gene Expression Omnibus (GEO) under accession number GSE91061. TPM was calculated from raw gene expression counts.

For the Liu anti-PD1, Van Allen anti-CTLA4, and Riaz anti-PD1 cohorts, the best overall response (BOR) to ICB was obtained from the original studies according to the RECIST (v1.1) criteria^41^. No clinical benefit (NCB) and durable clinical benefit (DCB) were defined as progressive disease (PD) and non-PD, respectively, as the best RECIST response. Patients with a mixed response (MR) or non-evaluable (NE) response were identified as having DCB if they remained in the programs for at least 6 months without disease progression, and NCB otherwise. Non-synonymous mutations were obtained from original studies^8,12,13^.

Transcriptomic data of the scRNA-seq cohort (n = 31) were acquired from the GEO database under the accession number GSE115978. Cell annotations were obtained from Jerby-Arnon et al.^14^. Twenty patients with $\ge$ 10 malignant cells were included in the analysis (post-immunotherapy-resistant, n = 8; untreated, n = 12).

### Tumor purity

An ESTIMATE score for each patient in the bulk RNA-seq cohort was inferred using ESTIMATE^42^ based on immune fractions and stromal fractions. Tumor purity was calculated using the following formula described by Yoshihara et al.^42^:

$$purity=\mathrm{cos}(0.6049872018+0.0001467884\times ESTIMATE\,score)$$

### APM gene expression correction

The Jerby-Arnon scRNA-seq cohort was used to calculate the gene-specific normalization factors of the set of APM genes participating in MHC class I antigen presentation, including *B2M*, *HLA-A*, *HLA-B*, *HLA-C*, *HLA-E*, *HLA-F*, *HLA-G*, *ERAP1*, *NLRC5*, *PSMB1*, *PSMB2*, *PSMB8*, *PSMB9*, *PSMB10*, *TAP1*, *TAP2*, and *TAPBP*. First, pseudobulk gene expression profiles at the patient level were calculated. The pseudobulk expression of each gene was calculated as the average number of TPM reads across all cells (excluding cells with undefined annotations from the original authors) of a patient. This patient-specific pseudobulk expression profile represents the bulk transcriptomic profile of a heterogeneous tumor sample. Pseudobulk expression profiles of malignant and non-malignant cells were calculated in the same manner. Tumor purity of each patient was represented as the fraction of malignant cells in the total cell population (excluding cells with undefined annotations). The normalization factor of each gene *x* was calculated as the ratio of the average non-malignant pseudobulk gene *x* expression to the average malignant pseudobulk gene *x* expression across all patients:

$${N}_{x}=\frac{\sum_{i}{\lambda }_{x,i}}{\sum_{i}{\gamma }_{x,i}}$$

where $i$ represents a patient, $\lambda$ is the expression profile of non-malignant cells, and $\gamma$ is the expression profile of malignant cells (represented by pseudobulk expression).

Next, we inferred APM gene expression in tumor cells from bulk RNA-seq expression profiles using normalization factors. Bulk RNA-seq expression of gene *x* was modelled as the weighted average of malignant and non-malignant expression by tumor purity:

$${\beta }_{x,i}={\gamma }_{x,i}\bullet {p}_{x,i}+{\lambda }_{x,i}\bullet (1-{p}_{x,i})$$

where $\beta$ is the bulk RNA-seq matrix and $p$ is the tumor purity. Under the assumption ${\lambda }_{x,i}={\gamma }_{x,i}\bullet {N}_{x}$, the tumor-specific expression of gene *x* is

$${\gamma }_{x,i}=\frac{{\beta }_{x,i}}{{p}_{x,i}+{N}_{x}-{N}_{x}\bullet {p}_{x,i}}$$

APM expression correction was applied to the Liu anti-PD1, Van Allen anti-CTLA4, and Riaz anti-PD1 bulk RNA-seq cohorts. The corrected expression values were used for all subsequent analyses.

### APM gene signature and unsupervised hierarchical clustering

The APM model is based on genes encoding MHC molecules, chaperones, immunoproteasomes, and other proteins involved in antigen processing and loading in the antigen presentation pathway. For the base model, we selected the classical and non-classical HLA class I genes (*HLA-A*, *HLA-B*, *HLA-C*, *HLA-E*, *HLA-G*, and *HLA-F*), class II genes (*HLA-DRA*, *HLA-DRB1*, *HLA-DQA1*, *HLA-DQB1*, *HLA-DQA2*, *HLA-DQB2*, *HLA-DPA1*, *HLA-DPB1*), MHC class I transactivator *NLRC5*, and class II transactivator *CIITA*.

Additional APM features, including proteasome genes (*PSME1*, *PSME2*, *PSMB8*, *PSMB9*, and *PSMB10*)^15,16^, *B2M* associated with the MHC I heavy chain, non-classical HLA II gene paralogues/pseudogenes (*HLA-DRB5* and *HLA-DRB6*)^16,43^, molecular chaperones assisting antigen loading (*HLA-DMA*, *HLA-DMB*, *HLA-DOA*, and *HLA-DOB*), and other APM genes (*ERAP1*, *TAPBP*, *TAP1*, and *TAP2*)^16^, were chosen for the forward-selection-based process. In particular, the powerset of these additional APM features was computed. The elements in the powerset were each added to the base model to perform unsupervised hierarchical clustering, and they were ranked based on the performance of the four-cluster APM model to independently predict patient survival using a log-rank test in the Liu anti-PD1 and Van Allen anti-CTLA4 cohorts (Supplementary Table S2). The top-ranked combination among all 65,536 combinations was added to the base model to form the featured APM gene signature (*HLA-A*, *HLA-B*, *HLA-C*, *HLA-E*, *HLA-G*, *HLA-F*, *HLA-DRA*, *HLA-DRB1*, *HLA-DQA1*, *HLA-DQB1*, *HLA-DQA2*, *HLA-DQB2*, *HLA-DPA1*, *HLA-DPB1*, *NLRC5*, *CIITA*, *PSME1*, *TAPBP*, *PSMB10*, *TAP2*, *HLA-DRB6*, *HLA-DMA*, *and HLA-DOB*), which was used for subsequent clustering and survival analysis in other cohorts.

### scRNA-seq quality control and clustering of immune cell types

To filter out low-quality reads, genes with fewer than five reads across all the cells were excluded. Genes that were upregulated under dissociation/stress conditions were excluded to avoid confounding factors during sample processing. For clustering, Seurat^44^ was used to perform SCTransform-based normalization, identify the top variable features, scale the features, and run principal component analysis (PCA). UMAP analysis was performed by Seurat to identify clusters. To define the specific T cell subsets, T cells annotated by Jerby-Arnon et al. (T-CD4, T-CD8, and T-Cell) were extracted. Dimensionality reduction was performed as previously described. The differential expression of canonical immune cell markers was computed using the FindAllMarkers function in Seurat.

### Gene set enrichment Analysis (GSEA) and single-sample gene set enrichment Analysis (ssGSEA)

Differential expression analysis of the bulk RNA-seq cohort was performed using DESeq2^45^. Differential expression analysis in the scRNA-seq cohort was performed using FindMarkers function in Seurat. Differentially expressed genes between the two cell populations were ranked using the signed log2 fold change. The fGSEA R package (RRID:SCR_020938) was used to compute pathway enrichment for the hallmark gene sets from the Molecular Signature Database (MSigDB) (http://www.gsea-msigdb.org/gsea/msigdb/collections.jsp), using the pre-ranked list of genes. The normalized enrichment score (NES) and false discovery rate (FDR) were used to evaluate differential enrichment of pathways.

ssGSEA projections were generated to transform the gene expression profiles of individual samples into gene set enrichment profiles. ssGSEA enrichment scores were calculated for hallmark gene sets from MSigDB using the corto R package^46^.

### Immune cell deconvolution

For the Liu, Van Allen, and Riaz cohorts, immune infiltrate estimates in each sample were inferred using CIBERSORTx^47^ with the RNA-seq TPM matrix and LM22 gene set in the absolute mode (https://cibersortx.stanford.edu). Quantile normalization was disabled.

### Classification

We used a multinomial logistic regression classifier to classify the tumors into one of the four clusters. The log-transformed TPM reads of the genes in the APM signature were first normalized within each cohort using housekeeping genes to correct for batch effects. Briefly, the housekeeping gene score for each patient was represented as the log average expression of the HG7 signature (*ACTB*, *GAPDH*, *UBC*, *HMBS*, *TBP*, *HPRT1*, *RPL13A*)^48^. The expression data of the genes in the APM signature were divided by the average housekeeping gene score within the cohort. For training, the normalized expression of selected APM genes and clustering results from the Liu anti-PD1 and Van Allen anti-CTLA4 cohorts were combined to train the logistic regression classifier with hyperparameters (solver = “saga,” C = 8, and max_iter = 1500) chosen based on the training set. A tenfold cross-validation was used to estimate the “out-of-bag” area under the curve (AUC). The performance of the classifier was evaluated using the mean cross-validation AUC. This process was repeated 1000 times.

To predict APM clusters in the Riaz validation cohort, HG7-normalized APM gene expression was used as a feature in the logistic regression classifier trained on the Liu and Van Allen cohorts. To predict the clusters in the Jerby-Arnon scRNA-seq cohort, the housekeeping gene score for each patient was calculated using patient-level pseudobulk expression profiles of all cells. The HG7-normalized pseudobulk expression of genes involved in MHC I and MHC II antigen presentation in malignant and non-malignant cells, respectively, was used as features in the logistic regression classifier.

### Statistics and survival analysis

All statistical analyses were performed using R version 4.0.4. Differences between the two groups were evaluated using two-sided Fisher’s exact test for categorical variables and two-sided Wilcoxon rank-sum test for continuous variables. Differences between groups with paired data were evaluated using the two-sided Wilcoxon signed-rank test. Differences across more than two groups were evaluated using Kruskal–Wallis one-way analysis of variance. Correlations between continuous variables were represented using Spearman’s rho coefficient. P-values were unadjusted.

The survival curves, the Kaplan–Meier estimator and nonparametric log-rank test were used to compare the survival curves. To calculate the hazard ratio and significance of Progression-Free Survival (PFS) and Overall Survival (OS) with multiple explanatory variables, multivariable Cox proportional hazards modeling was used. To select the APM gene signature, the log-rank test was used to evaluate the performance of the model.

### Ethical compliance

No human subjects were involved in the study.

## Supplementary Information

Supplementary Information 1.

Supplementary Information 2.

Supplementary Information 3.

Supplementary Information 4.

Supplementary Information 5.

Supplementary Information 6.

Supplementary Information 7.

Supplementary Information 8.

Supplementary Information 9.

## Data Availability

The current study analyzed existing, publicly available cohorts downloaded from dbGaP and GEO under accession numbers (phs000452.v3.p1, phs000452.v2.p1, GSE91061, GSE115978). Gene expression and downstream analysis data are available within the article and its supplementary data files. The datasets used and/or analyzed during the current study are available from the corresponding author on reasonable request.

## Code availability

All original codes have been deposited at https://github.com/KarchinLab/APM-Clustering-ICB-Manuscript.
