# Supplementary material for: Clustering by antigen-presenting genes reveals immune landscapes and predicts response to checkpoint immunotherapy
Source: Sci Rep. 2023 Jan 18;13:950. doi: 10.1038/s41598-023-28167-1 (PMC9849403; doi:10.1038/s41598-023-28167-1)
Supplement: Supplementary file 1 — Supplementary Information 1. [file 41598_2023_28167_MOESM1_ESM.docx]

Clustering by antigen-presenting genes reveals immune landscapes and predicts response to checkpoint immunotherapy

Xutong Gong and Rachel Karchin


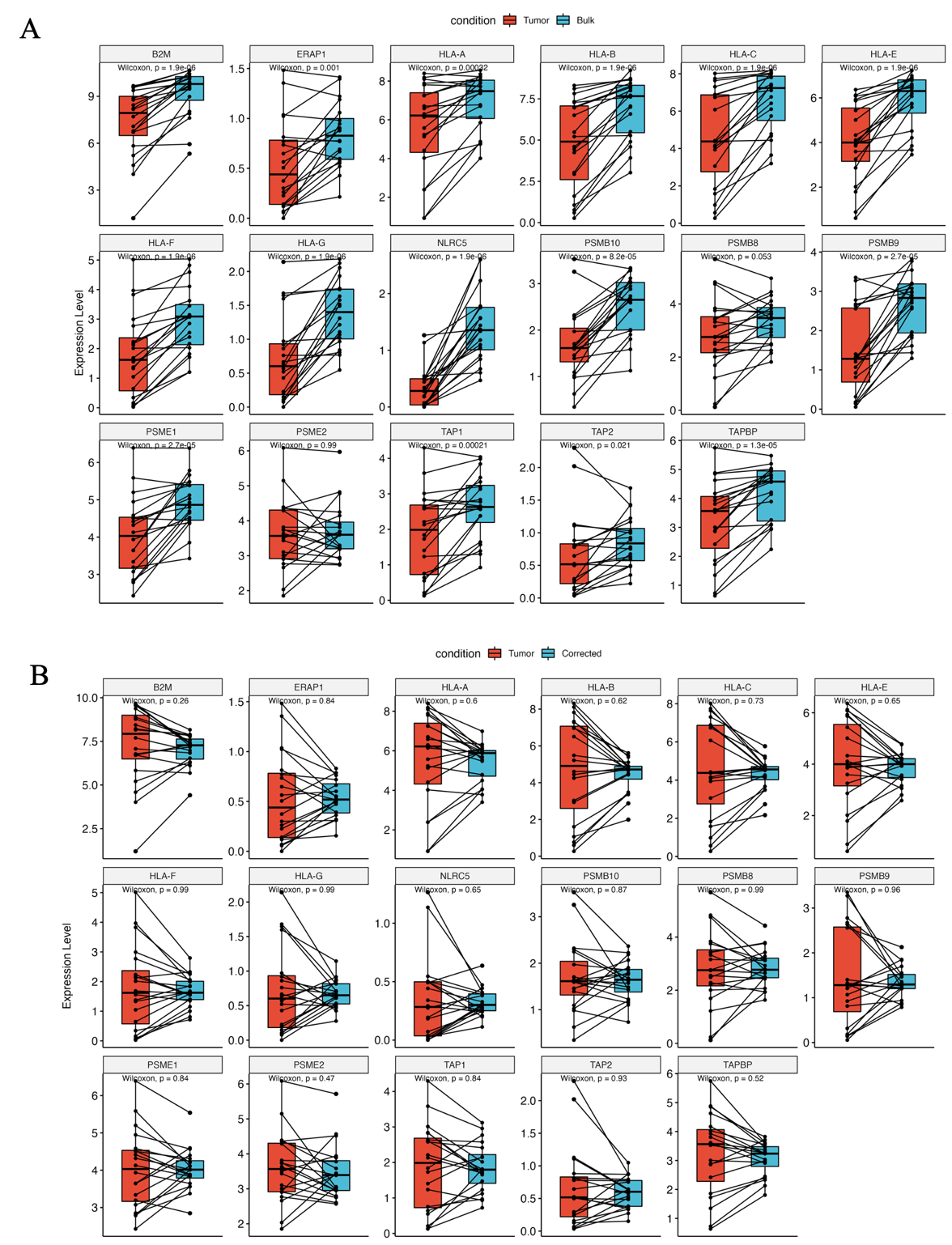


**Figure S1. APM expression correction. A,** Comparison of the tumor-specific and bulk expression of MHC class I and associated genes. Patient-level pseudobulk expression profiles of malignant cells and all cells (malignant + non-malignant) were used to represent tumor-specific and bulk expression, respectively. Boxes in the boxplots represent interquartile ranges and vertical lines represent 5^th^-95^th^ percentile ranges. Significance was computed by Wilcoxon signed-rank test. **B,** Same as in (**A**), but for tumor-specific and corrected bulk expression.

**
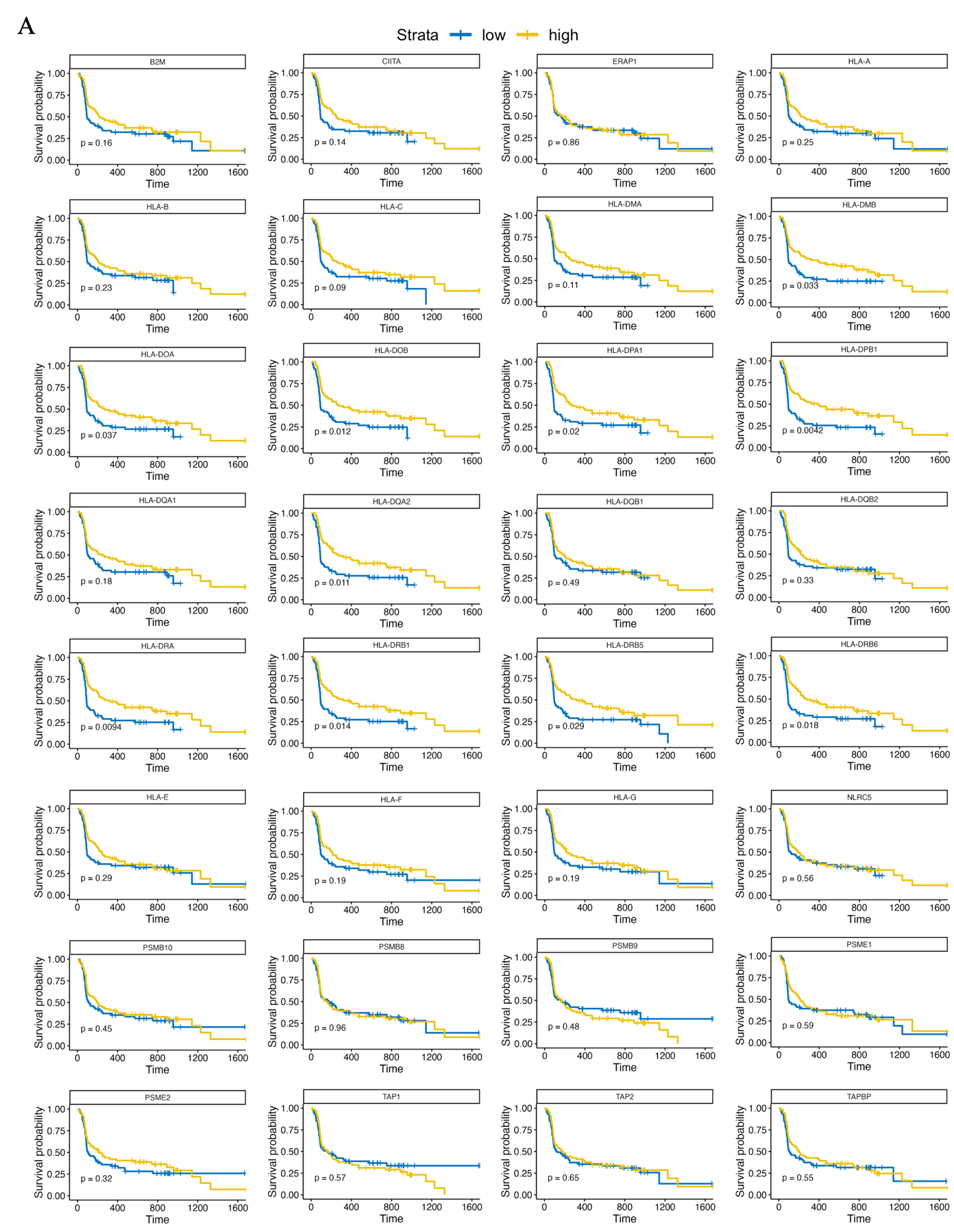
**

**
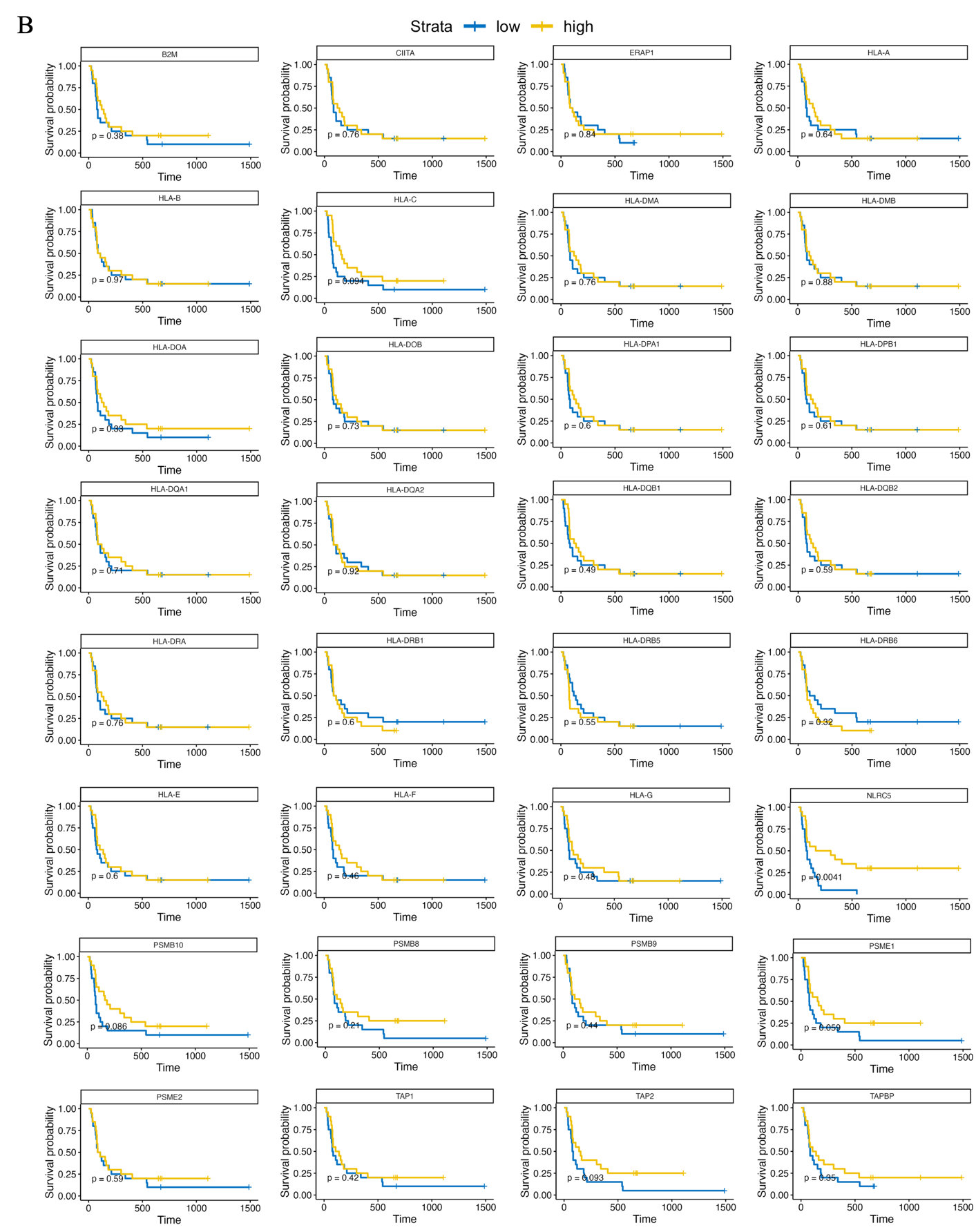
**

**Figure S2. APM gene expression correlates with varied survival outcomes. A-B**, Progression-free survival stratified by expression of APM genes (split by the median) as labeled in the Liu anti-PD1 cohort (**A**) and Van Allen anti-CTLA4 cohort (**B**). P-values were computed by Log-rank test.

**
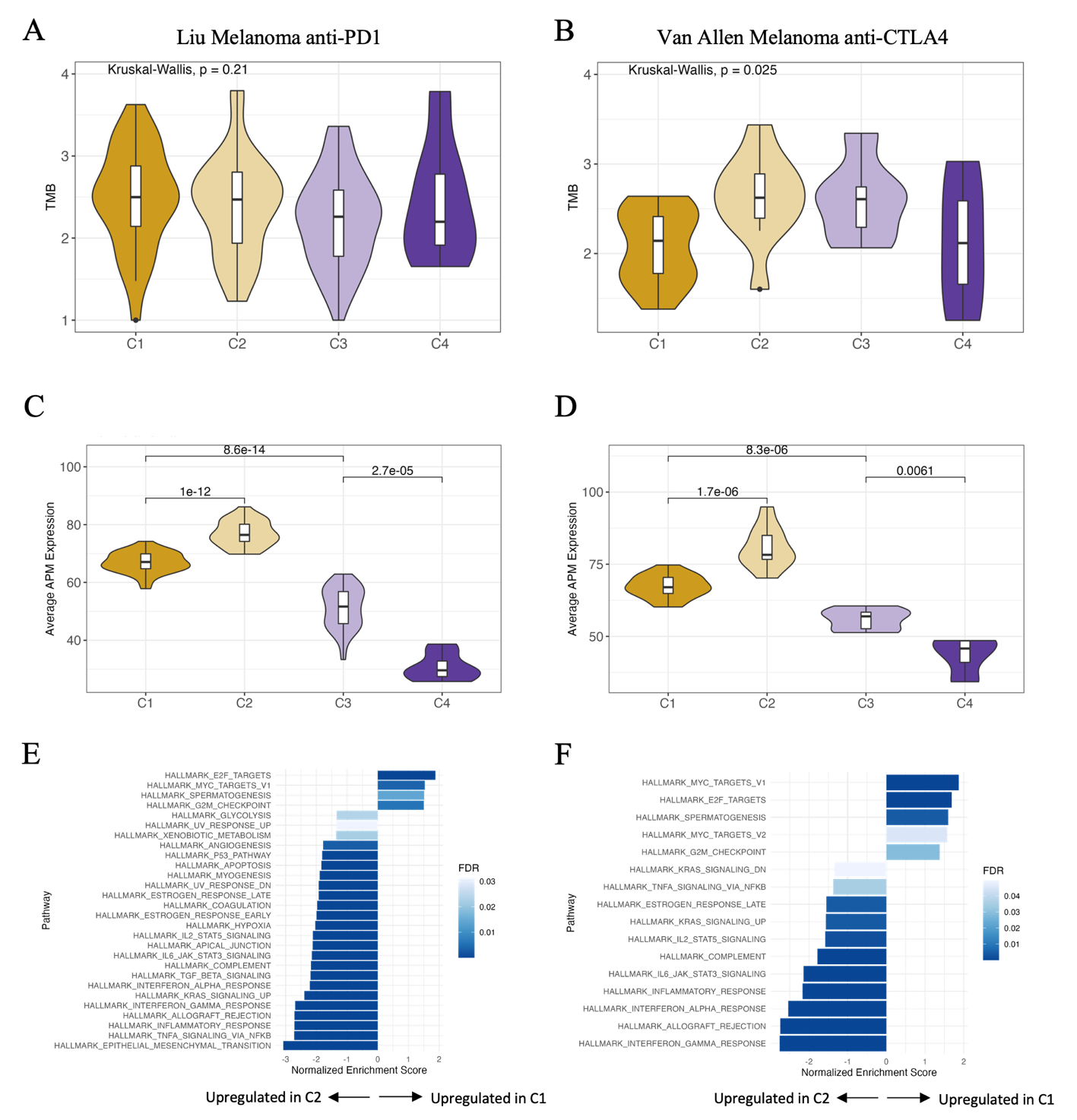
**

**
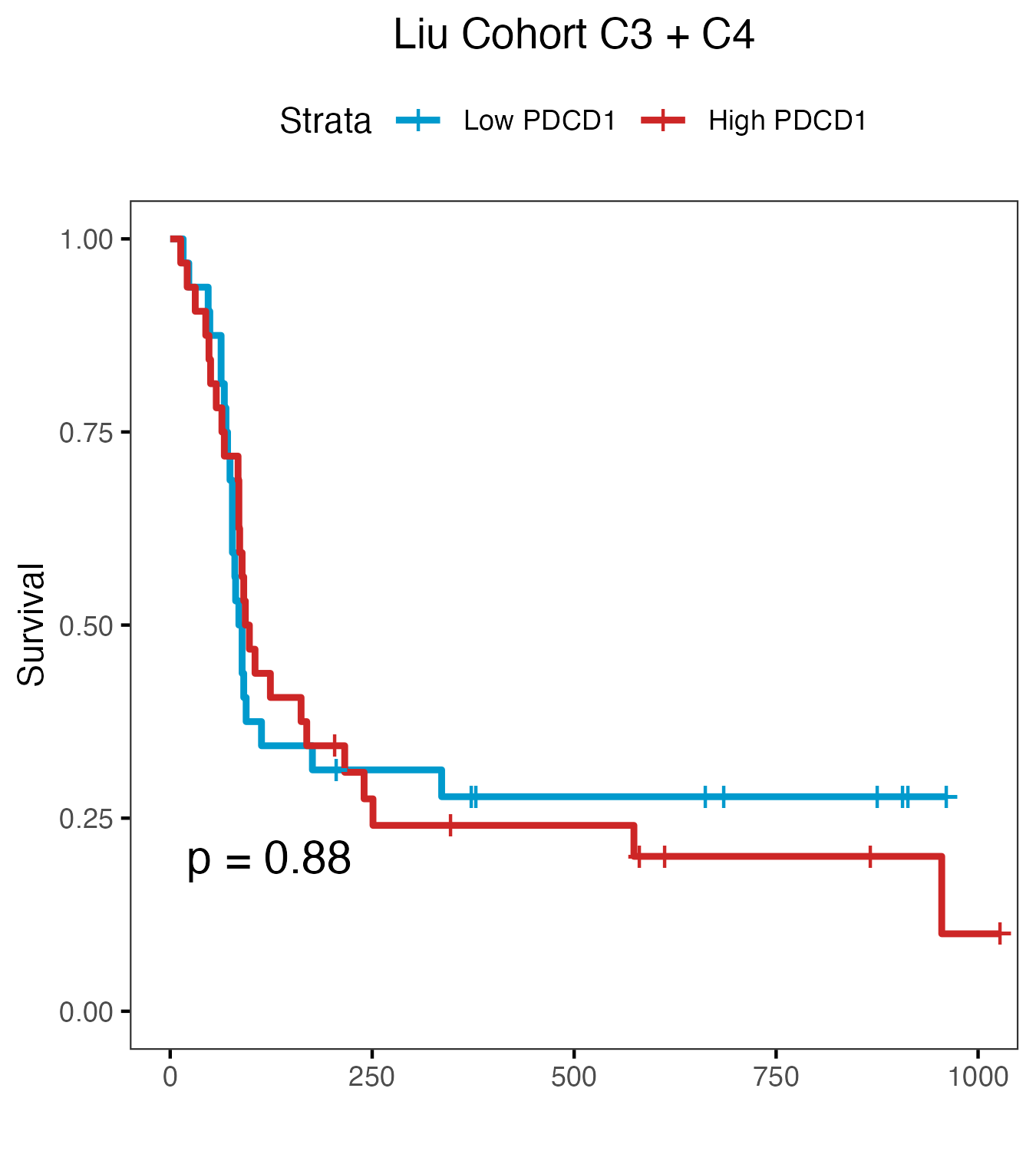

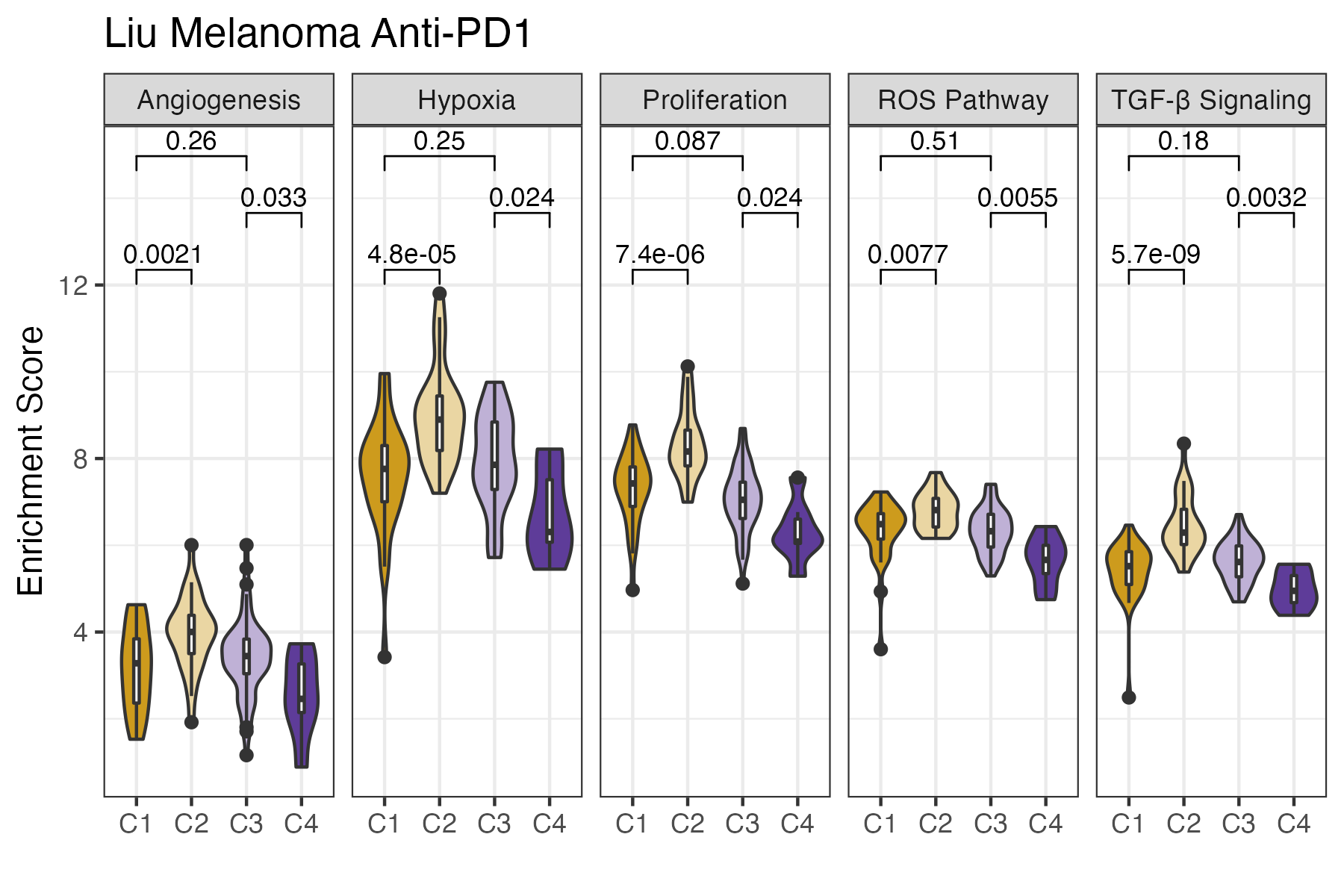

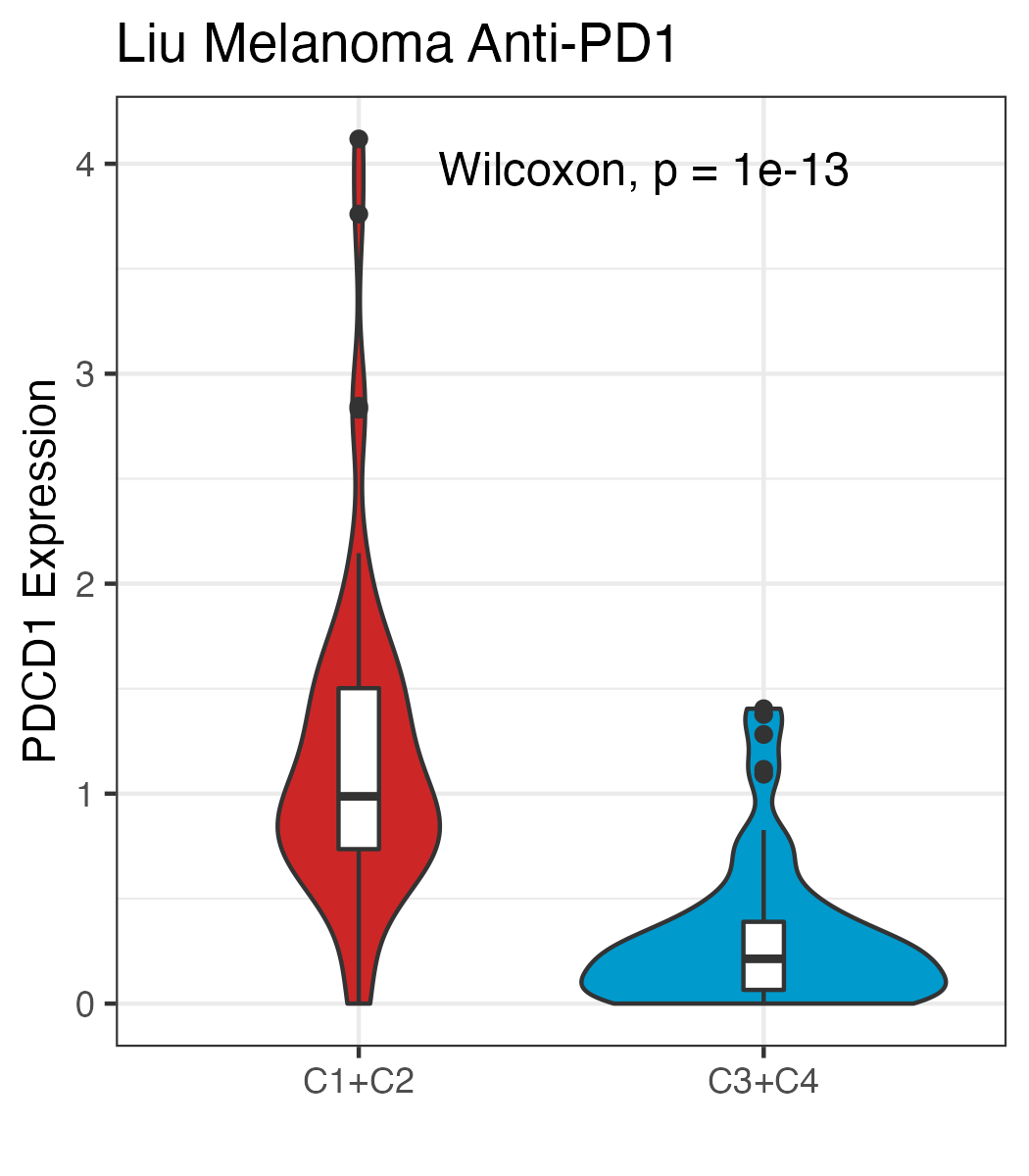

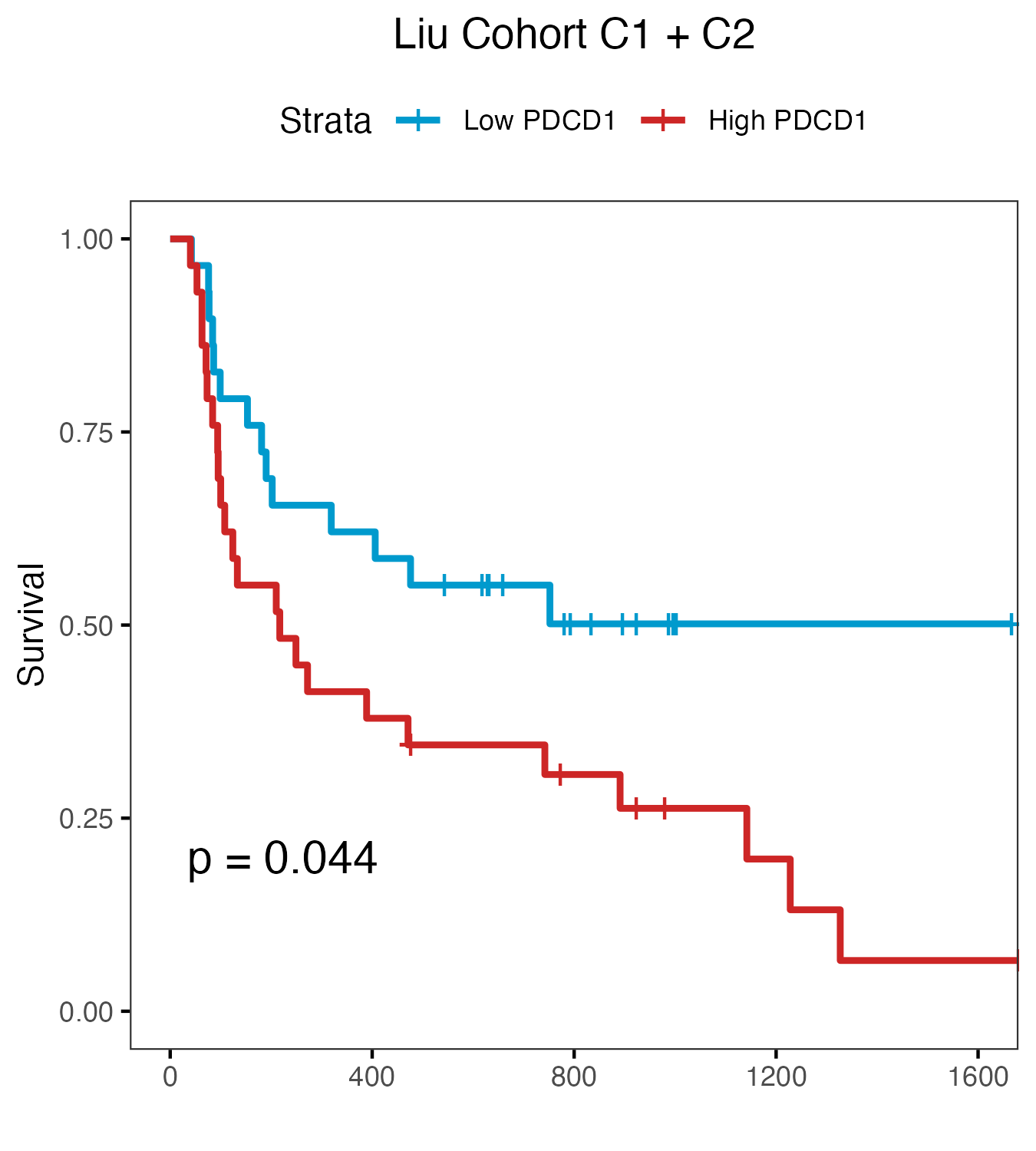
**

I

H

J

G

**Figure S3. Tumor intrinsic characteristics across clusters.** **A-B**, TMB across clusters in the Liu cohort (**A**) and Van Allen cohort (**B**). Boxes in the violin plots represent interquartile ranges and vertical lines represent 5^th^-95^th^ percentile ranges. Significance was computed by Kruskal-Wallis test. **C-D**, APM expression, represented as the log average expression of 23 APM genes used in clustering, across clusters in the Liu cohort (**C**) and Van Allen cohort (**D**). Significance between the clusters was computed by Wilcoxon rank-sum test. **E-F**, Pathway enrichment analysis computed by GSEA in tumors clustered as C1 and C2 in the Liu cohort (**E**) and Van Allen cohort (**F**). The hallmark gene category annotated by the Molecular Signature Database (MSigDB) was used. Pathways with positive normalized enrichment scores (NES) were enriched in C1 tumors, and those with negative NES were enriched in C2 tumors. Colors represent false discovery rate (FDR). Pathways with FDR $\leq$ 0.05 are shown. **G**, ssGSEA enrichment scores of the hallmark gene sets annotated by the MSigDB across clusters in the Liu cohort. Significance between the clusters was computed by Wilcoxon rank-sum test. **H**, Progression-free survival (PFS) by *PDCD1* expression (split by the median) in C1 and C2 tumors in the Liu cohort. P-value was computed by Log-rank test. **I,** Same as in (**H**), but in C3 and C4 tumors. **J**, *PDCD1* expression across hot tumors (C1 and C2) and cold tumors (C3 and C4). Significance was computed by Wilcoxon rank-sum test.


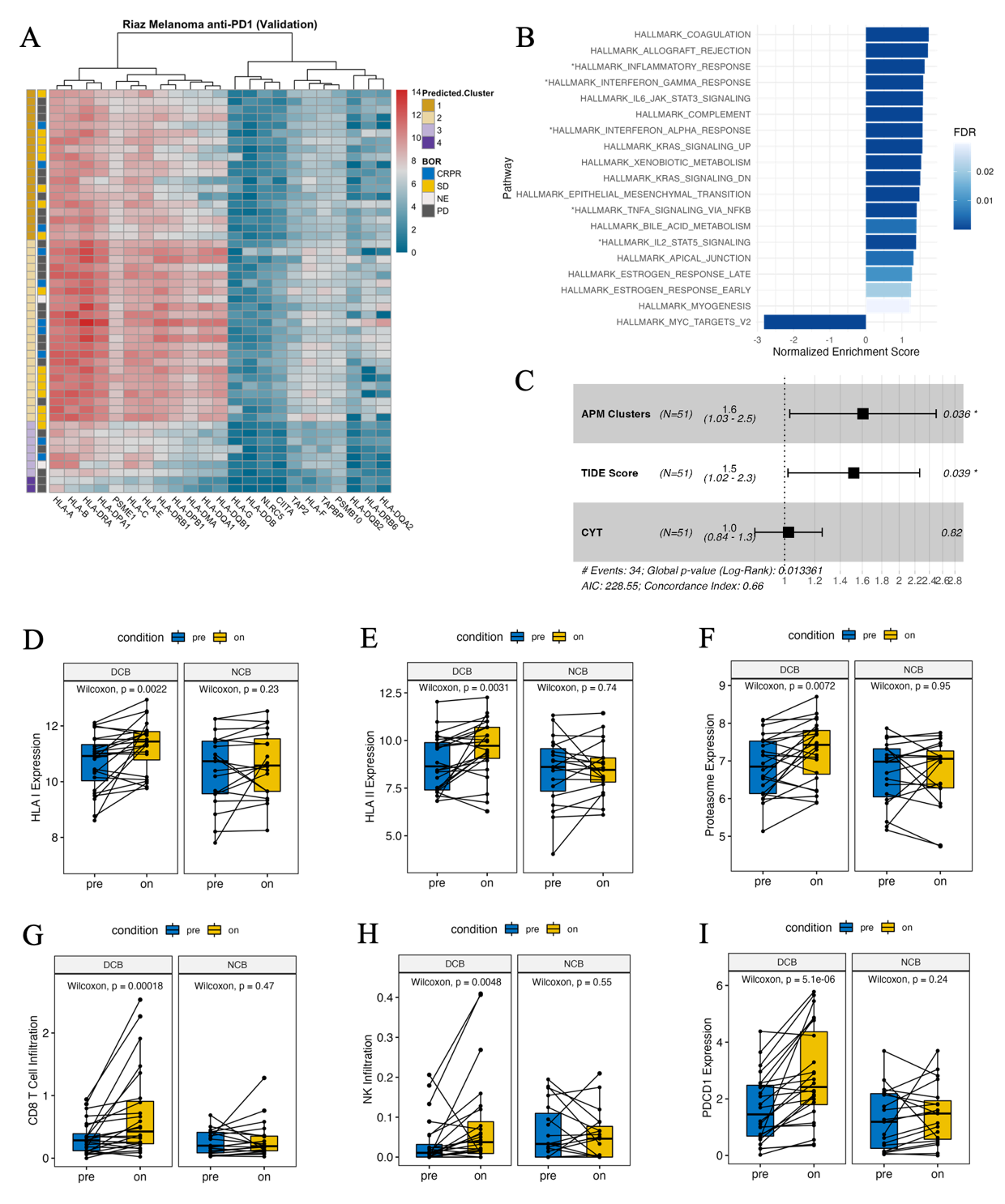


**Figure S4. APM upregulation is critical for triggering durable response to ICB.** **A**, Heatmap of APM gene expression across the predicted APM C1-C4 in the Riaz anti-PD1 cohort (validation). pheatmap function in pheatmap version 1.0.12 (https://www.rdocumentation.org/packages/pheatmap/versions/1.0.12) was used to generate the heatmaps. Each row represents a patient, and each column represents a gene. Color scale represents gene expression as log transformed transcripts per million (TPM) reads. BOR: best of response; CRPR: complete/partial response; SD: stable disease; MR: mixed response; PD: progressive disease. **B,** Pathway enrichment analysis computed by GSEA. The hallmark gene category annotated by the MSigDB was used. Pathways with positive normalized enrichment scores (NES) were enriched in C1 and C2 tumors, and those with negative NES were enriched in C3 and C4 tumors. Asterisks denote proinflammatory pathways. Colors represent false discovery rate (FDR). Pathways with FDR $\leq$ 0.05 are shown. **C**, Multivariate Cox proportional hazards model comparing the APM model to published prognostic signatures. APM clusters (C1=1, C2=2, C3=3, C4=4), CYT, and the TIDE scores are continuous variables. Hazard ratios (HR), 95% confidence intervals (CI), and p-values are shown. **D-F**, Comparison of APM expression, including HLA class I (**D**), class II (**E**), and proteasome genes (**F**), in pre- and on-treatment tumors between DCB and NCB. Boxes in the boxplots represent interquartile ranges and vertical lines represent 5^th^-95^th^ percentile ranges. Significance was computed by Wilcoxon signed-rank test. **G-H**, Same as **D-F**, but for CD8 T cell (**G**) and NK cell infiltration (**H**). **I**, Same as **D-F**, but for *PDCD1* expression.


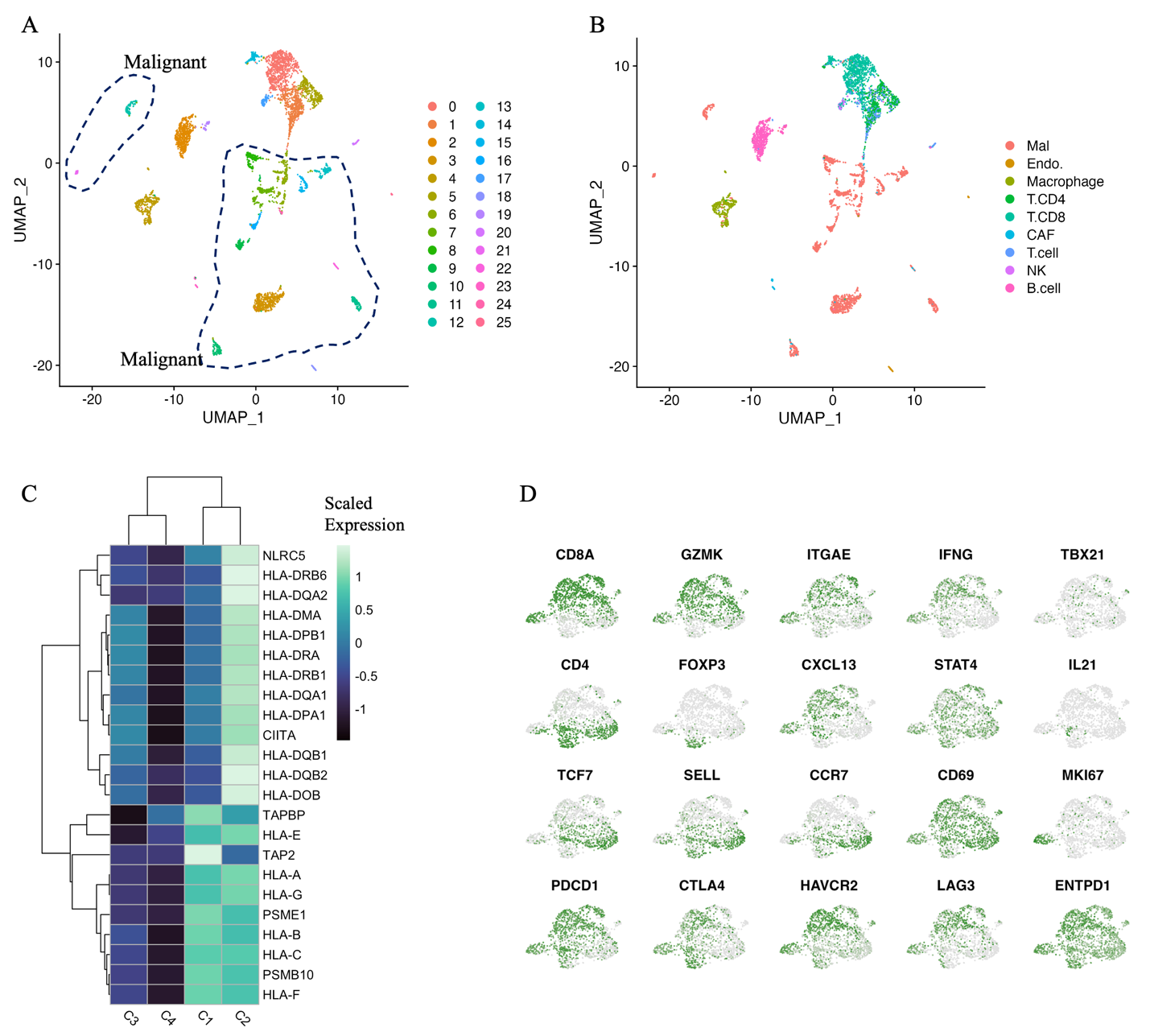


**Figure S5. Cluster annotations of UMAP analyses in the scRNA-seq cohort.** **A,** UMAP analysis of all cells in the Jerby-Arnon scRNA-seq cohort. 26 clusters were identified. **B,** Distribution of cell types annotated by Jerby-Arnon et al. on the UMAP plot of all cells. Each color represents a cell type as indicated. Mal: malignant, Endo.: endothelial, CAF: cancer-associated fibroblasts. **C,** Pseudobulk expression profiles of APM genes on the APM cluster-level. pheatmap function in pheatmap version 1.0.12 (https://www.rdocumentation.org/packages/pheatmap/versions/1.0.12) was used to generate the heatmaps. Color scale represents scaled pseudobulk expression. **D,** Expression of T cell canonical, subset-selective, and checkpoints/exhaustion markers on the UMAP plot of the T cell population.

**Table S1. Numbers of post-treatment vs. untreated patients across APM clusters in the scRNA-seq cohort.**

|  | C1 | C2 | C3 | C4 |
| --- | --- | --- | --- | --- |
| Untreated | 5 | 1 | 5 | 1 |
| Post-immunotherapy (resistant) | 0 | 0 | 8 | 0 |
