# Supplementary material for: Clustering by antigen-presenting genes reveals immune landscapes and predicts response to checkpoint immunotherapy
Source: Sci Rep. 2023 Jan 18;13:950. doi: 10.1038/s41598-023-28167-1 (PMC9849403; doi:10.1038/s41598-023-28167-1)
Supplement: Supplementary file 2 — Supplementary Information 2. [file 41598_2023_28167_MOESM2_ESM.docx]

**Supplemental Tables**

**Table S2.** A ranked list of all feature combinations and their associated survival significance by hierarchical clustering in the discovery cohorts.

**Table S3.** APM clustering results for patients in the bulk and scRNA-seq cohorts.

**Table S4.** Corrected APM expression in the bulk RNA-seq cohorts and pseudobulk APM expression in the scRNA-seq cohort.

**Table S5.** Cell type clustering and differential gene expression analysis in the scRNA-seq cohort.

**Table S6.** Gene sets of immune activities.

**Table S7.** TIDE scores and cytolytic scores (CYT) in the Riaz cohort.

**Table S8.** Differential gene expression analysis between C1-C2 and C3-C4 in the bulk RNA-seq cohorts.
